# Mapping End-Stage Renal Disease (ESRD): Spatial Variations on Small Area Level in Northern France, and Association with Deprivation

**DOI:** 10.1371/journal.pone.0110132

**Published:** 2014-11-03

**Authors:** Florent Occelli, Annabelle Deram, Michaël Génin, Christian Noël, Damien Cuny, François Glowacki, on behalf of the Néphronor Network

**Affiliations:** 1 EA 4483, Université Lille Nord de France, Faculté de Pharmacie de Lille, Lille, France; 2 Faculté Ingénierie et Management de la Santé (ILIS), Loos, France; 3 EA 2694, Université Lille Nord de France, Faculté de Médecine pôle Recherche, Lille, France; 4 Service de Néphrologie, Hopital Huriez, CHRU de Lille, Lille, France; 5 Réseau Néphronor, Hôpital Huriez, CHRU de Lille, Lille, France; University of Perugia, Italy

## Abstract

**Background:**

Strong geographic variations in the incidence of end-stage renal disease (ESRD) are observed in developed countries. The reasons for these variations are unknown. They may reflect regional inequalities in the population's sociodemographic characteristics, related diseases, or medical practice patterns. In France, at the district level, the highest incidence rates have been found in the Nord-Pas-de-Calais region. This area, with a high population density and homogeneous healthcare provision, represents a geographic situation which is quite suitable for the study, over small areas, of spatial disparities in the incidence of ESRD, together with their correlation with a deprivation index and other risk factors.

**Methods:**

The Renal Epidemiology and Information Network is a national registry, which lists all ESRD patients in France. All cases included in the Nord-Pas-de-Calais registry between 2005 and 2011 were extracted. Adjusted and smoothed standardized incidence ratio (SIR) was calculated for each of the 170 cantons, thanks to a hierarchical Bayesian model. The correlation between ESRD incidence and deprivation was assessed using the quintiles of Townsend index. Relative risk (RR) and credible intervals (CI) were estimated for each quintile.

**Results:**

Significant spatial disparities in ESRD incidence were found within the Nord-Pas-de-Calais region. The sex- and age-adjusted, smoothed SIRs varied from 0.66 to 1.64. Although no correlation is found with diabetic or vascular nephropathy, the smoothed SIRs are correlated with the Townsend index (RR: 1.18, 95% CI [1.00–1.34] for Q2; 1.28, 95% CI [1.11–1.47] for Q3; 1.30, 95% CI [1.14–1.51] for Q4; 1.44, 95% CI [1.32–1.74] for Q5).

**Conclusion:**

For the first time at this aggregation level in France, this study reveals significant geographic differences in ESRD incidence. Unlike the time of renal replacement care, deprivation is certainly a determinant in this phenomenon. This association is probably independent of the patients' financial ability to gain access to healthcare.

## Introduction

In developed countries, the burdens of End-Stage Renal Disease (ESRD) and Renal Replacement Therapy (RRT) were continuously growing and now stabilize since the early 2000s. In sharp contrast, incidence rates are still growing in developing countries [1]–[4]. At the scale of a country, strong geographic variations in the incidence of treated ESRD have been observed [5]–[13]. In metropolitan France, the crude incidence rate of RRT also varies widely, from 80.4 to 238.6 per million inhabitants (pmi) in 71 districts in 2006–2007 [14]. Rates were highest in the northeast and south and lowest in the west and east. In 2008–2009, it varies from 85.8 to 225.5 pmi in 85 districts, with higher rates in north-east and southern France and lower rates in the western part [15].

The reasons for these variations remain elusive. They may result from inter-regional variations in the population's sociodemographic characteristics [6], [10], [15]–[17], from other related diseases such as diabetes and cardiovascular diseases [8], [15], [18]–[20], or merely reflect differences in the timing of dialysis initiation [14], [21] and geographic distance from healthcare facilities [22].

On a smaller scale, the spatial variability of treated ESRD and its relationship to risk factors has also been assessed for US counties or census tracts [23]–[28], UK wards [22] and Australian postcodes [17]. There is no such equivalent in France. Such a spatial approach, made at the scale of homogeneous populations over a territory with similar health practices, may provide an improved knowledge of ESRD patterns, allowing an explanation to be found for these disparities. Such an outcome would then lead to a better understanding of environmental assumptions [29]–[31].

The French Renal Epidemiology and Information Network (REIN) is a national Chronic Kidney Disease (CKD) registry, which lists all patients who initiated ESRD treatment since 2002, and is currently available in 22 regions of metropolitan France [32]. Among these, the Nord-Pas-de-Calais, a small region with 4 million inhabitants, has the highest ESRD incidence, with a standardized rate of 207 new cases pmi in 2012, as opposed to 154 new cases pmi for all of France [33]. The homogeneous healthcare access (median travel-time to dialysis units: 15 min, and>45 min for only 0.46% of the patients [33]), the large number of cases and the associated population density make the geographic situation of this region quite suitable for the study of disparities in ESRD incidence in small areas, and their correlation with sociodemographic status, as well as the quality of environmental media.

The aim of this study was to analyze the spatial variations of ESRD incidence over small areas, and to analyze the correlation between geographic variability and social discrepancies (assessed using the Townsend deprivation index). The study focuses on the Nord-Pas-de-Calais, for the period between 2005 and 2011.

## Materials and Methods

### Study area and sources of data

The Nord-Pas-de-Calais region has a surface area of 12 481 km^2^ with approximately 4 033 000 inhabitants, including both rural, industrial and urban regions. The region's 170 cantons (a French small administrative unit) were used to represent distinct spatial units. These are referenced by the National Institute of Statistics and Economic Studies (INSEE), and in 2009 had an average population of 23 725 (extremes ranging from 4 991 to 226 827) and an average surface area of 73 km^2^ (extremes ranging from 2 to 258 km^2^).

Cases were defined as all incident patients requiring RRT registered in the REIN registry (patients were registered on the first day of RRT) in the Nord-Pas-de-Calais region, from January 2005 to December 2011 [34]. For the purpose of this study, all patients were grouped into cantons according to the postcode of their residence, determined at the time of their first RRT. They were ranked by sex and 5-year age group. The following characteristics of the patients were also collected: primary kidney disease, number of visits to a nephrologist in the year preceding treatment, estimated Glomerular Filtration Rate (eGFR) by Modification of Diet in Renal Disease (MDRD) formula at the time of dialysis initiation [35].

Demographic and socioeconomic data were extracted from the 2009 national population census, provided by the INSEE. The population data was also ranked by sex and 5-year age groups. For the Townsend deprivation index calculation, the 2009 French census output area file was matched to each patient's canton of residence. The score was computed from 4 census variables: percentages of non-owner-occupied households, unemployment, household overcrowding, and absence of access to a motor vehicle [36].

### Ethics Statement

This study was conducted in accordance with Commission Nationale de l'Informatique et des Libertés (CNIL) and Comité consultatif sur le traitement de l'information en matière de recherche dans le domaine de la santé (CCTIR).

### Statistical analysis

#### Incidence rates and deprivation index

Firstly, the male and female crude incidence rates were estimated for the 170 cantons, by means of direct standardization. Crude incidence is the number of new patients divided by the total population at risk during the study period. The SIR, defined as the ratio of the number of observed cases to the expected number of cases, computed using indirect standardization, was then determined for each canton. Notice that the SIR is the maximum likelihood estimate of 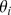, the true relative risk (RR) associated with canton 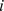. Significant SIRs have a 95% credible interval, which does not contain the value 1. The method used to calculate the incidence rate denominators assumed the population in a given canton to remain constant over the study period.

On the basis of our registry, the proportion of cases with diabetic and/or vascular nephropathy was calculated for each canton. The Townsend index for each canton was classified into quintiles, with the first quintile (Q1) corresponding to the least deprived cantons, and the fifth quintile (Q5) corresponding to the most deprived cantons.

The Pearson correlation was used to assess the correlation between the logarithm of the SIR and the eGFR.

#### Spatial analysis

The centroid of each canton, defined by its geographical center (longitude and latitude), was used for the spatial analysis. In order to take the instability resulting from low frequencies and spatial autocorrelation effect into account, the SIR was smoothed using the hierarchical Bayesian model with three levels, proposed by Besag et al. [37]. At the first level, the observed number of cases in the 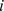 ^th^ canton 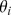 is assumed to be Poisson distributed,
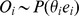
with a mean 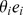, where 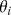 is the RR associated with canton 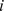, and 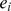, the expected number of cases, considered as constant and calculated by means of indirect standardization. At the second level the logarithm of 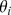 is modeled as the sum of two random effects, the first, 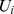, corresponding to the unstructured spatial heterogeneity and the second, 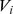, describing the correlation between the neighboring cantons (sharing a common boundary):



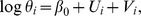



where 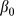 corresponds to overall level of the RR across the study region. The random effect 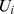 is distributed as a normal distribution of null mean and variance 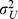:
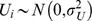
and the random effect 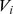 is modeled using the conditional autoregressive (CAR) model:



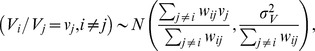
where 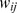 = 1 if the canton 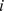 and canton 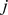 are adjacent and 0 otherwise. At the third level, the variances of the two random effects 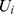 and 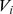, 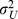 and 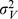, have a non-informative gamma prior distribution as suggested by Bernadinelli et al. [38].

In order to analyze the association between ESRD and the deprivation index, the quintiles of the latter were introduced at the second level of the hierarchical model as follow:



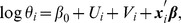
where 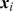 corresponds to the vector of covariates and 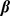 the vector of the associated coefficients. In order to take into account the quintiles of the deprivation index, we introduced four dummy variables 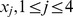 corresponding to the second, third, fourth and fifth quintile. The first quintile has been taken as reference. Each 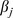 has a non-informative normal prior distribution as suggested by Lawson et al. [39].

The models were fitted using Markov Chain Monte Carlo methods with 25 000 iterations, following a burning step involving 5 000 iterations. All of the calculations were made using the WinBUGS Software [40], and the maps were produced using the ArcGIS 10.1 software (http://www.esri.com). All statistical analyses were considered significant at the 0.05 type 1 error.

## Results

**Figure 1:**
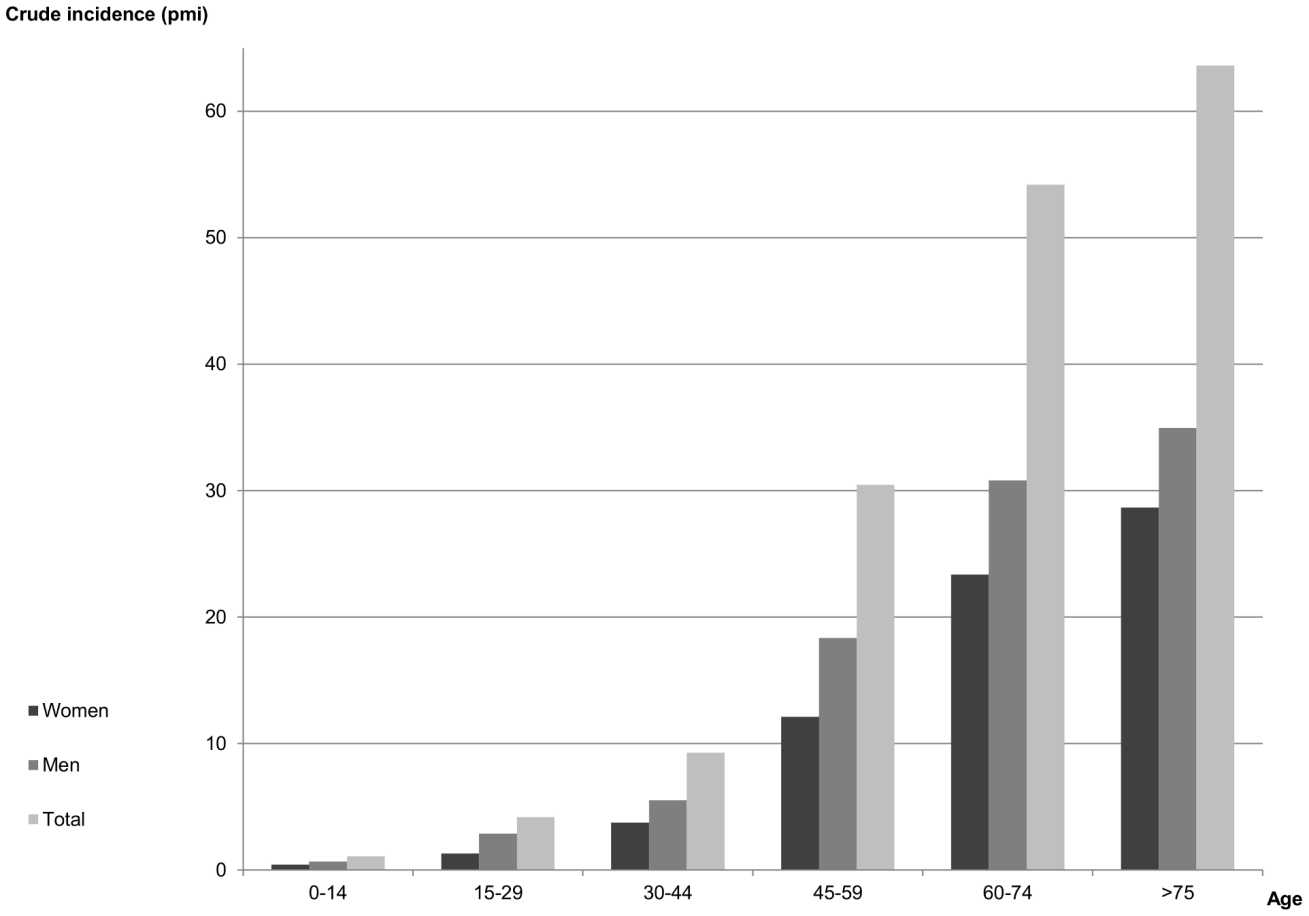
Crude incidence rate of ESRD (pmi) by age and gender.

The study included 4 597 patients (57.2% men and 42.8% women), who began an RRT between 2005 and 2011 in the Nord-Pas-de-Calais. The overall crude annual incidence rate was 163 pmi for both sexes, 193 pmi for men and 135 pmi for women. Strong disparities were observed over the 170 cantons, ranging from 38 to 432 pmi for both sexes combined, and from 0 to 424 pmi for men and 0 to 439 pmi for women (Table 1). As expected, the crude incidence rate increases sharply with age, and males have a relatively higher proportion of ESRD incidence (Fig. 1). The median age of incidents is 69 years for men, 72 years for women, and 71 years for both sexes combined.

**Table 1:** 

	Mean	SD	Min	Q1	Median	Q3	Max
**Number of patients**	27	26	2	12	21	32	219
**Total crude incidence (pmi)**	158	53	38	124	157	186	432
**Men crude incidence (pmi)**	187	72	0	144	182	230	424
**Women crude incidence (pmi)**	130	61	0	89	123	169	439
**Smoothed SIR**	0.96	0.20	0.66	0.81	0.92	1.07	1.64
**Townsend index**	0.00	3.71	−6.01	−2.91	−0.97	2.82	10.80
**Diabetic nephropathy (%)**	27.5	11.7	0.0	20.0	27.6	33.3	75.0
**Vascular nephropathy (%)**	24.4	12.5	0.0	16.7	23.7	32.0	100.0
**eGFR (ml/min/1.73 m^2^)**	9.05	1.46	5.59	8.05	8.92	9.84	14.40

**Figure 2:**
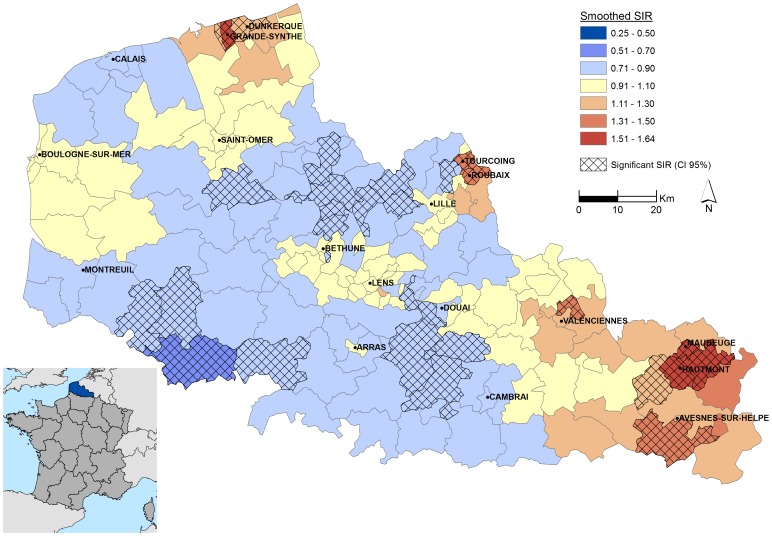
Smoothed SIRs of ESRD by cantons, 2005–2011.

The smoothed SIRs vary among cantons, from 0.66 to 1.64 (Table 1), and there is a significant spatial variability of SIR within the Nord-Pas-de-Calais region (Fig. 2). There are four areas with significantly high incidence rates. In the South-East, Maubeuge, Hautmont and Avesnes-sur-Helpe are rural cantons, were SIRs are respectively 1.64, 95% CI [1.32–2.01], 1.50, 95% CI [1.18–1.88] and 1.39, 95% CI [1.05–1.79]. Northern part of Valenciennes (SIR: 1.38, 95% CI [1.12–1.68]) is more urbanized and includes several industrial activities. The Agglomeration of Roubaix, Tourcoing and Wattrelos (1.48, 95% CI [1.26–1.73], 1.32 95% CI [1.13–1.54] and 1.33, 95% CI [1.07–1.63] respectively) is a densely populated urban area (≈230,000 inhabitants). In northern, SIRs are respectively 1.55, 95% CI [1.18–2.01] and 1.27, 95% CI [1.10–1.47] in Grande-Synthe and Dunkerque, which are important industrial zones, surrounded by a densely populated urban area (≈220,000 inhabitants). Three areas with significantly low incidence rates are observed. They mostly include rural areas, such as in South-Western region (SIR: 0.66, 95% CI [0.46–0.89]).

Before assessing the relationship between deprivation and these spatial disparities, the influence of early referral dialysis was examined. Early dialysis initiation was determined by measuring the median eGFR for each canton (Table 1). Although it is significant (p<0.05), the Pearson coefficient (R = 0.23) indicates a very weak positive correlation with the smoothed SIRs.

Disparities are observed in the proportions of diabetic or vascular nephropathy occurring in the different cantons within the region (Table 1). These are not correlated with the smoothed SIRs. The RR is 1.31, 95% CI [0.87–1.97] for diabetic nephropathy and 0.98, 95% CI [0.65–1.48] for vascular nephropathy.

**Figure 3:**
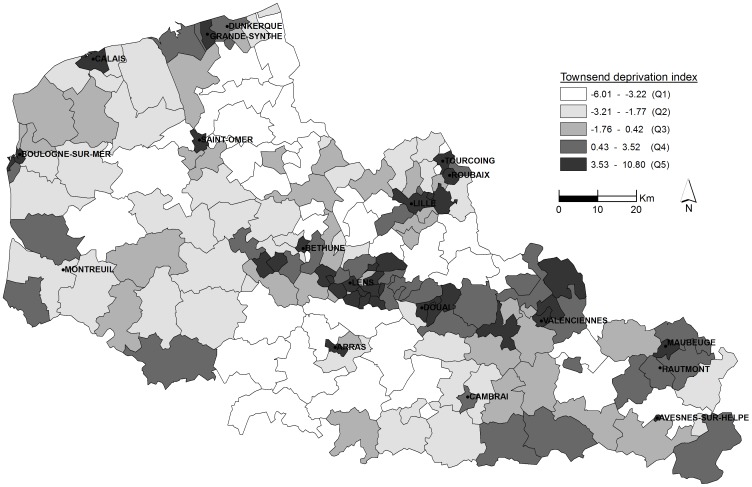
Spatial distribution of Townsend deprivation index by cantons, 2009.

**Figure 4:**
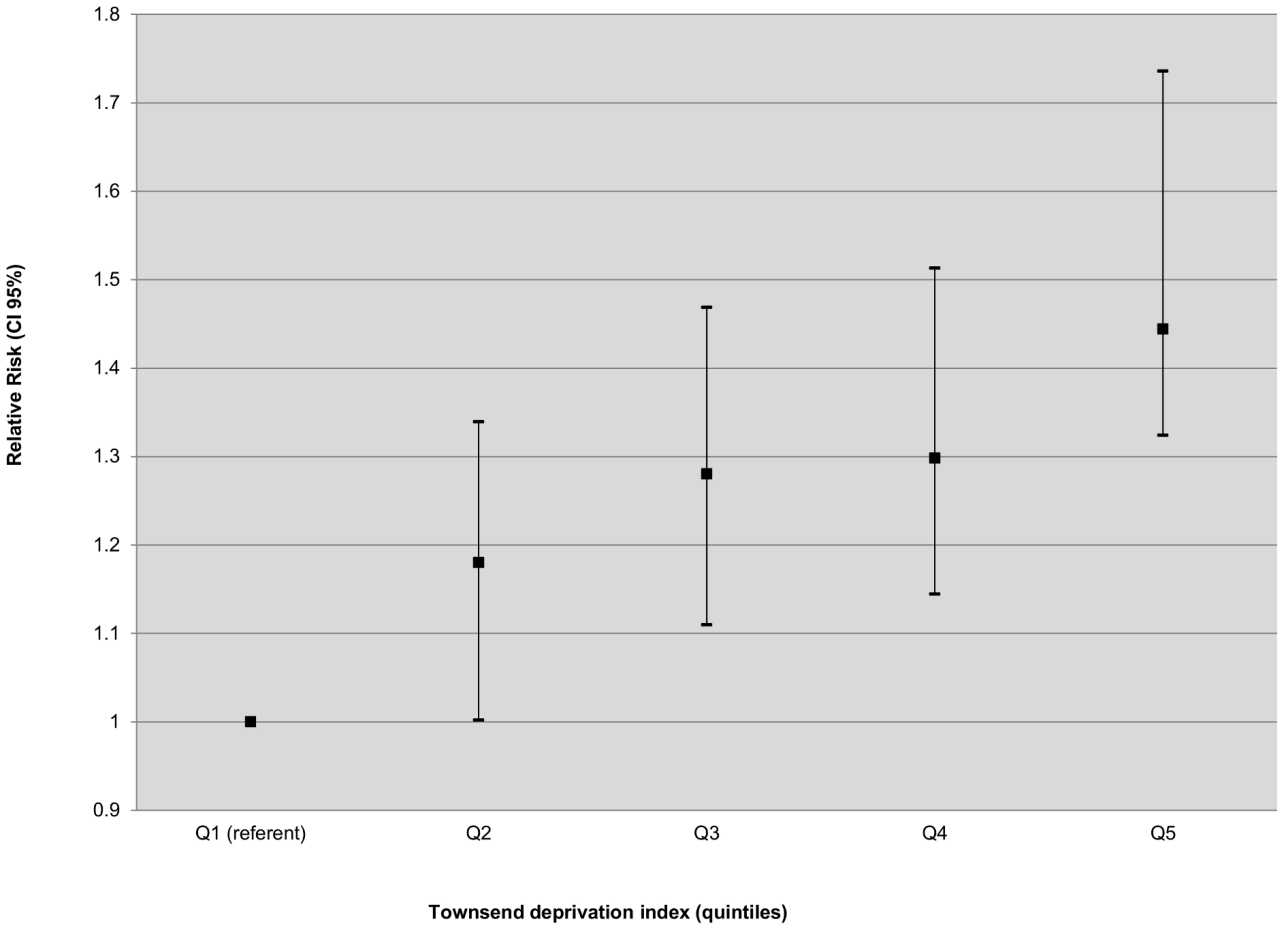
Relative risk (95% credible interval) of ESRD by Townsend quintile.

The Townsend deprivation index varies strongly within the region (Table 1, Fig. 3), and there is a significant correlation between smoothed SIRs and the Townsend index quintiles. With Q1 taken as a reference, the relative risk (RR) of RRT was assessed for each level of deprivation (Fig. 4). Higher levels of deprivation are associated with an increase in RR: 1.18, 95% CI [1.00–1.34] for Q2, 1.28, 95% CI [1.11–1.47] for Q3, 1.30, 95% CI [1.14–1.51] for Q4 and 1.44, 95% CI [1.32–1.74] for Q5. In addition, the residential Townsend index attributed to each collected case was compared with late referral, estimated by means of the number of visits to a nephrologist in the year preceding treatment. No correlation is found (p = 0.66).

**Figure 5:**
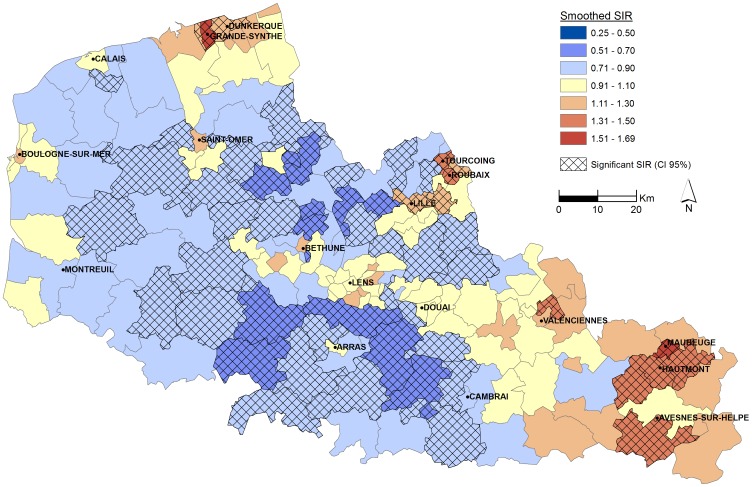
Smoothed SIRs of ESRD by cantons, 2005–2011, adjusted to the Townsend index covariable, 2009.

Analysis of the geographic patterns of the smoothed SIRs, adjusted to the Townsend covariable, still reveal three areas of significantly high risk in the north and south-east of the region, in the Lille metropolis and the agglomerations of Roubaix, Tourcoing and Wattrelos. Two significantly low-risk areas are also observed in the south-west of the region and the area between Saint-Omer and Lille (Fig. 5). The corresponding smoothed SIRs vary among cantons, from 0.66 to 1.69.

## Discussion

In this study, significant spatial disparities in ESRD incidence are revealed in the 170 cantons of the Nord-Pas-de-Calais region of France. Although such disparities have recently been observed among districts, they had never been seen at this spatial resolution in France. These results are consistent with variations observed at similar scales in other countries [6], [17], [22], [28].

Several factors were analyzed, in an attempt to explain this phenomenon. It should be noted that any ecological correlations were made at the level of geographical areas, and not individuals. As a consequence, although causality cannot be assumed, etiological hypotheses can be proposed [41].

In France, Couchoud et al. [14] found that the intensity of healthcare has a substantial impact on RRT incidence at the level of individual districts. Concerning the Nord-Pas-de-Calais region, a significant, but weak association was found between the median eGFR and the smoothed SIRs. Earlier timing of dialysis initiation has a negligible contribution to spatial disparities in the incidence of ESRD within this region. This result confirms the presence of uniform medical practice throughout this territory.

The spatial heterogeneity of ESRD is not related to the incidence of diabetic or vascular nephropathies. This result means that cantons with high incidence rates are not associated with an overincidence of diabetic or vascular nephropathies. The cases developing such CKD were not the cause of the observed phenomenon. However, several studies have identified ecological relationships between RRT incidence and the prevalence of diabetics or cardio-vascular diseases [8], [15], [18]–[20]. As no data was available concerning the prevalence of these diseases at the scale of each canton, the present study data was used as a proxy, to determine the proportion of diabetic and vascular nephropathies in each canton.

On the other hand, deprivation is clearly associated with a higher ESRD incidence, and wealth with a lower ESRD incidence. These findings are consistent with results observed on a larger scale in France, since Couchoud et al. [15] revealed a positive relationship between 82 districts, whatever the socio-economic factors used. The patterns shown here are similar to those found in other studies, which focused on deprivation indicators over larger heterogeneous geographic areas. Caskey et al. [42] identified a correlation between RRT incidence and a country's macroeconomic factors, such as gross domestic product (GDP) per capita, percentage of GDP spent on health care, and dialysis facility reimbursement rate relative to GDP. Although Ward et al. [16] highlighted a greater incidence of ESRD in patients living in Zoning Improvement Plan (ZIP) areas with a lower composite socioeconomic score, this trend was not uniform for all primary renal diseases. In the case of US counties, the incidence rate of treated ESRD has been shown to be inversely related to the level of income [25]. For similar, highly homogenous small area units, Grace et al. [17] recently found a decreasing incidence of RRT with increasing area advantage in Australia. In the UK, deprivation is found to be a determinant of geographical variations in RRT, between wards or enumeration districts [6], [43]. Furthermore, Volkova et al. [28] have revealed a strong correlation between incidence rates and neighborhood poverty, corresponding to populations living below the poverty level, for the case of the census tracts of Georgia, North Carolina, or South Carolina. Although the Townsend index is criticized for its urban view of deprivation, it is nevertheless widely used. Furthermore, some recently developed indexes have been shown to be strongly correlated with this one [44], [45].

In agreement with other studies, the assumption is made that deprivation is an obstacle to prevention, and that it supports the progression of CKD to ESRD [15], [16]. To substantiate this hypothesis, the influence of late referral from collected cases was assessed, by determining the number of visits to a nephrologist during the year preceding treatment. There was no significant correlation between this number and the Townsend index. Patients who were treated later do not live primarily in disadvantaged townships. Moreover, in France, access to healthcare and medical insurance coverage does not discriminate against poverty. The medical or hospital fees associated with CKD treatment are also completely covered by public health insurance. It is thus possible that the correlation between deprivation and ESRD incidence is not related to an individual's financial capacity to access healthcare. Other more relevant factors associated with deprivation, such as health literacy, acculturation or trust in healthcare providers, could explain these variations. This is supported by Lora et al. [46], who report that lower levels of health literacy and acculturation are associated with differences in knowledge, attitude, and behavior, that may contribute to a poor outcome in patients with CKD.

In this paper, it is shown that SIR mapping can be used to highlight global spatial heterogeneities in ESRD incidence. Although this method is needed to reveal spatial patterns of interest, it cannot detect significant atypical spatial and space-time clusters in terms of ESRD incidence. Spatial and space-time scan statistics [47], [48] should thus be used to test for the presence of ESRD clusters, and to identify their location in space and time.

When the SIR map is adjusted to the Townsend covariable, areas of significantly high incidence still exist. These are not explained in the present study. Other etiological assumptions such as environmental contamination should be assessed in the future. As it includes industrial, urban and agricultural territories, the Nord-Pas-de-Calais region is suitable for such a study. This should be conducted at the level of small areas, to avoid a dilution of the spatial variations characterizing nearby, heterogeneous populations.

## Conclusion

In developed countries, significant variations in ESRD incidence are observed over small areas [6], [17], [28]. The present study shows that this is also the case in France. Within the Nord-Pas-de-Calais territory, which has homogeneous healthcare provision, this phenomenon can be partially explained by deprivation. However, since access to healthcare is universal in France, and in the case of serious illnesses such as ESRD, this access is not affected by an individual's financial well-being, other deprivation-related factors may explain the observed correlation. Moreover, these disparities are not related to a specific type of medical practice, related to the initiation of dialysis. The SIR can be used as a geographic tool, for decision-making in the management of dialysis units and the definition of prevention campaigns at local scales. Even when the deprivation factor is taken into account, spatial disparities in ESRD incidence remain, suggesting that environmental factors such as suspected heavy metal still play a significant role [29]–[31]. We plan to investigate this aspect in future studies.
